# Correction: Periaqueductal efferents to dopamine and GABA neurons of the VTA

**DOI:** 10.1371/journal.pone.0219476

**Published:** 2019-07-03

**Authors:** Niels R. Ntamati, Meaghan Creed, Ridouane Achargui, Christian Lüscher

The following information is missing from the Funding statement: Funding was provided by the European Research Council (ERC Advanced grant MeSSI).
